# Numerical Modeling of Mechanical Behavior for Buried Steel Pipelines Crossing Subsidence Strata

**DOI:** 10.1371/journal.pone.0130459

**Published:** 2015-06-23

**Authors:** J. Zhang, Z. Liang, C. J. Han

**Affiliations:** School of Mechatronic Engineering, Southwest Petroleum University, Chengdu, China; Beijing University of Posts and Telecommunications, CHINA

## Abstract

This paper addresses the mechanical behavior of buried steel pipeline crossing subsidence strata. The investigation is based on numerical simulation of the nonlinear response of the pipeline-soil system through finite element method, considering large strain and displacement, inelastic material behavior of buried pipeline and the surrounding soil, as well as contact and friction on the pipeline-soil interface. Effects of key parameters on the mechanical behavior of buried pipeline were investigated, such as strata subsidence, diameter-thickness ratio, buried depth, internal pressure, friction coefficient and soil properties. The results show that the maximum strain appears on the outer transition subsidence section of the pipeline, and its cross section is concave shaped. With the increasing of strata subsidence and diameter-thickness ratio, the out of roundness, longitudinal strain and equivalent plastic strain increase gradually. With the buried depth increasing, the deflection, out of roundness and strain of the pipeline decrease. Internal pressure and friction coefficient have little effect on the deflection of buried pipeline. Out of roundness is reduced and the strain is increased gradually with the increasing of internal pressure. The physical properties of soil have a great influence on the mechanical properties of buried pipeline. The results from the present study can be used for the development of optimization design and preventive maintenance for buried steel pipelines.

## Introduction

Buried pipelines are the main transportation tools for oil and gas. They are greatly influenced by the strata. Especially, pipelines are prone to bending deformation, nudity, dangling and rupture [1] when they cross the loess strata, hills, coalmine gob areas and other subsidence strata. Therefore, it is very important to research the mechanical behaviors of buried pipelines crossing subsidence strata. This paper focuses on the structural response of continuous buried pipeline crossing subsidence strata. Those pipelines are subjected to an imposed deformation pattern, associated with the development of high stresses and strains in critical locations, which are well beyond the elastic range of pipeline material and may cause pipeline failure. In particular, high tensile stresses may cause fracture of the pipeline wall, especially at defected locations or welds [2], where high compression stress may cause buckling, either in the form of beam-type instability.

To assess pipeline strength against an imposed subsidence strata, state of stress within the pipeline should be calculated for the imposed deformation. Continuing the pioneering paper by Newmark and Hall [3], Kennedy [4] developed an analytical model considering a non-uniform friction interface between pipeline and soil. The cylindrical shell theory was put forward by Ariman and Muleski [5]. A simplify analytical formula of buried pipeline under sedimentation was established by Gao and Fang based on the elastic foundation [6]. Wang and Yeh [7] improved this methodology accounting for pipeline bending stiffness, whereas Vougiouskas [8] considered both horizontal and faults vertical movement and numerically analyzed buried pipelines as elastic beams. Liang [9] assessed the deformation and stress distribution of buried pipelines based on the elastic foundation beam model. The behavior of buried steel pipeline subjected to excessive ground-induced deformation has received significant attention in recent years. It has been recognized that, apart from geometric and mechanical properties of the steel pipeline. Site conditions may have a strong influence on pipeline response, and pipeline-soil interaction should be taken into account [10]. Shang [11] analyzed the deformation and stress of buried pipelines under collapse section by using Winkler model. Yuan [12] established the deformation model of submarine pipeline under a landslide. Wang [13] proposed the simplified evaluation formula of maximum stress and strain for buried pipeline crossing mining subsidence area. Liu [14] analyzed the responses of buried pipeline subjected to ground settlement by using equivalent spring boundary.

The present paper established the mechanical model of buried pipeline deformation crossing subsidence strata. The mechanical behaviors of buried pipeline from two steel grades (X65 and X80) with respect to appropriate performance criteria were examined. Considering the pipeline-soil interaction, effects of subsidence, diameter-thickness ratio, buried depth, internal pressure, friction coefficient and soil properties on the mechanical behavior of buried pipeline were investigated.

### Pipeline characteristics under subsidence strata

**Fig 1:**
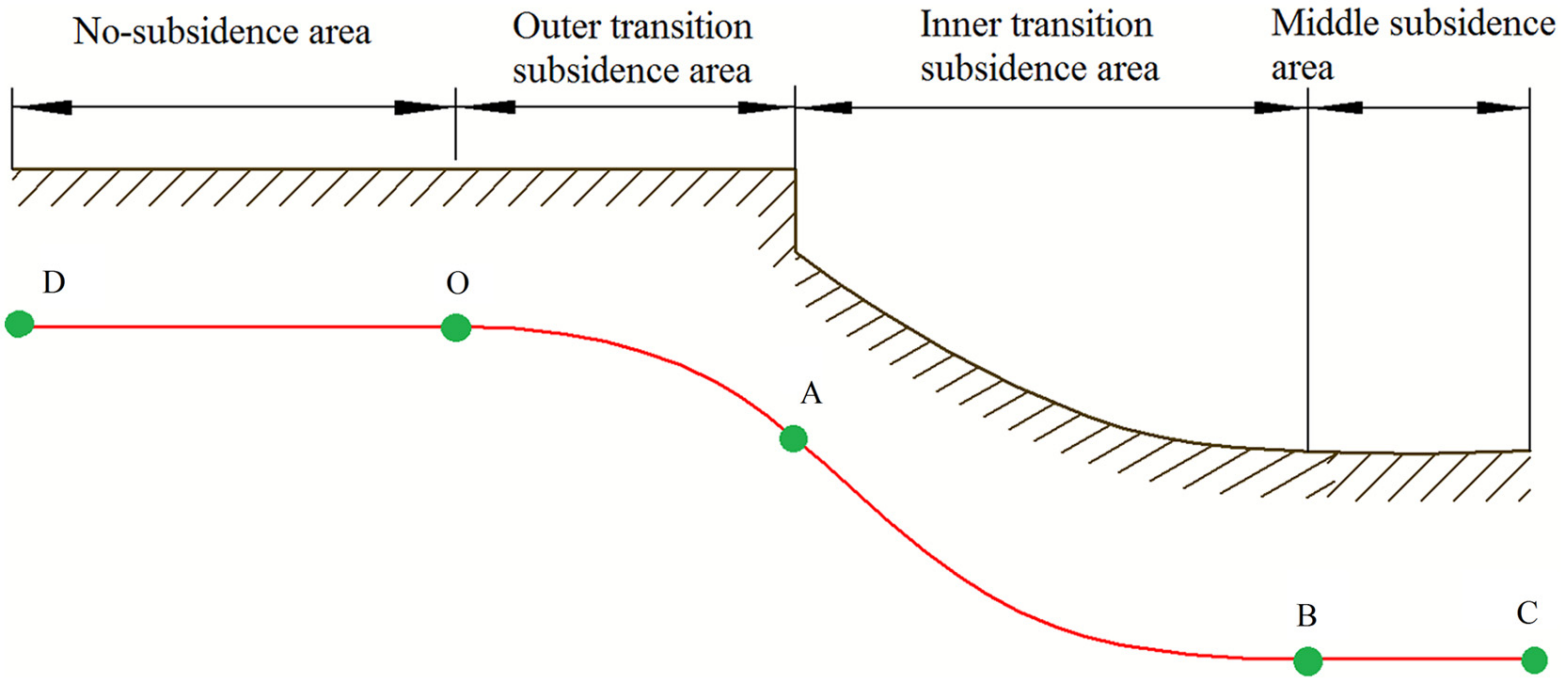
Schematic diagram of strata subsidence and pipeline bending deformation. The red line DC represents the buried pipeline, and it is divided into four sections according to the strata deformation.

As the collapse of coalmine gob areas or caverns, or subsidence of lower strata under action of seepage, the upper strata begin to sink. Then, the basic structure was formed as showed in Fig 1. According to characteristics of the subsidence area, it can be divided into four areas. The first one is middle subsidence area corresponding to BC segment of pipeline. The second one is inner transition subsidence area corresponding to AB segment of pipeline. The third one is outer transition subsidence area corresponding to OA segment of pipeline. The last one is no-subsidence area corresponding to DO segment of pipeline.

Middle subsidence area is located in the middle of subsidence area, surface subsidence is relatively uniform and the settlement volume is the biggest. Inner transition subsidence area is the transition between middle subsidence and outer middle subsidence, the surface subsidence is relatively non-uniform, a concave appears and compression deformation generates. The surface subsidence of outer subsidence area is non-uniform, and a convex appears. There is no surface subsidence in the non-subsidence area.

### Numerical calculation model

It is very difficult to analyze the buckling destruction of pipeline by using cable model and beam model [15]. When the large deformation appears on the cross section of pipeline, superposition principle can't be used for the interaction of longitudinal strain and bending strain for pipeline is a thin shell structure. There may be residual stress and stress concentration for the pipeline, therefore, it is difficult to solve the pipeline response by the analytic method. The finite element method is more suitable.

Structural response of steel pipeline under subsidence strata is examined numerically, using the general purpose finite element program ABAQUS. The nonlinear material behavior of the steel pipeline and the surrounding soil, the interaction between the soil and buried pipeline, as well as the distortion of the pipeline cross-section and deformation of the surrounding soil are modeled in a rigorous manner, so that the pipeline performance criteria is evaluated with a high-level of accuracy.

**Fig 2:**
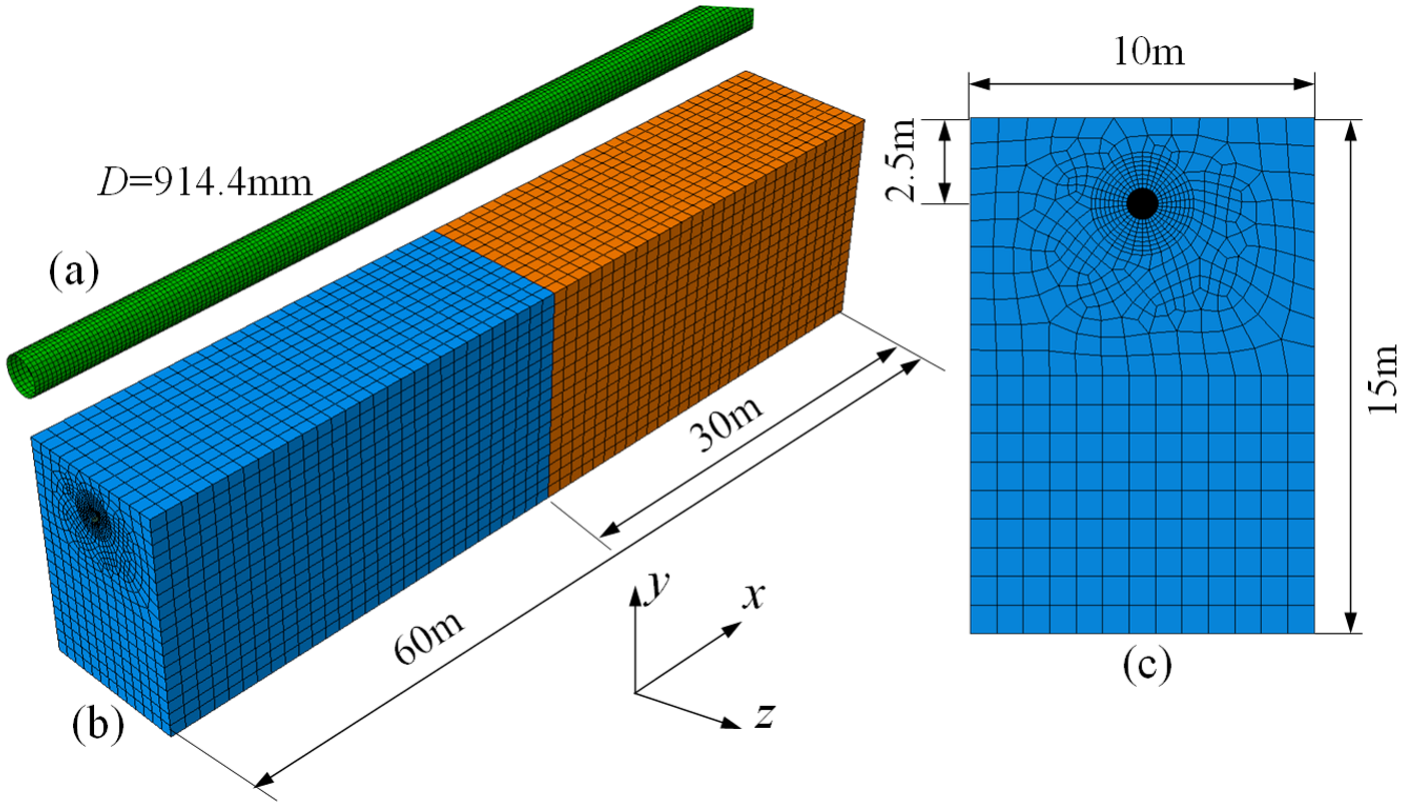
Finite element models of the soil and buried pipeline. (a) Finite element model of the buried pipeline. (b) Finite element model of the strata, and it is divided into two blocks. (c) Cross section of the whole model.

Fig 2a shows the buried pipeline mesh and Fig 2c depicts the mesh of soil in *yz* plane. The pipeline is embedded in an elongated soil prism along the *x* axis as shown in Fig 2b. Four-node reduced-integration shell elements (S4R) are employed to model the buried pipeline, and eight-node reduced-integration elements (C3D8R) are used to simulate the surrounding soil. Buried depth is chosen equal to about 2 times pipeline diameter, which is in accordance with pipeline engineering practice [16]. The soil length in *x* direction is equal to at least 60 pipeline diameters, where dimensions in directions *y* and *z* equal to 15 and 10 times the pipeline diameter respectively. A total of 30 shell elements around the cylinder circumference in this central part have been found to be adequate to achieve convergence of the solution, whereas the size of the shell elements in the longitudinal direction has been chosen equal to 1/20 of the pipeline outer diameter *D*.

The subsidence interface plane divides the soil in two blocks of equal size (Fig 2b). The analysis is conducted in two steps as follows, gravity loading is applied firstly. Then subsidence displacement *w* is imposed, which increases gradually. The nodes on the bottom boundary planes of the first block (soil nodes) remain fixed in the *y* directions. A uniform vertical displacement due to subsidence is imposed at bottom nodes of the second block. Symmetry constraint is applied to the end surface of the second block. In addition, all nodes on the end boundary plane of the first block are fixed with respect to horizontal displacement (*x* direction). For pressure pipelines, an intermediate step of internal pressure application is considered (after the application of gravity and before subsidence displacement is activated).

A large-strain von Mises plasticity model with isotropic hardening is employed for the steel pipeline material. Mechanical behavior of soil material is described through an elastic-perfectly plastic Mohr-Coulomb constitutive model, characterized by cohesion *c*, friction angle *ϕ*, elastic modulus *E*, and Poisson's ratio *ν*. The dilation angle is assumed equal to zero for cases considered in this paper.

The interface between outer surface of the pipeline and the surrounding soil is simulated with a contact algorithm, which allows separation of the pipeline and soil, and accounts for interface friction, through an appropriate friction coefficient *μ*. In the majority of results reported in this study, *μ* is considered equal to 0.3.

## Results and Discussions

Numerical results are obtained for X65 and X80 steel pipelines. The pipeline diameter is 0.9144m (36 in), which is a typical size for oil and gas transmission pipeline. The pipeline wall thickness *t* is considered equal to 8mm. The pipeline-soil model has dimensions 60m×15m×10m in directions *x*, *y*, *z* respectively.

**Fig 3:**
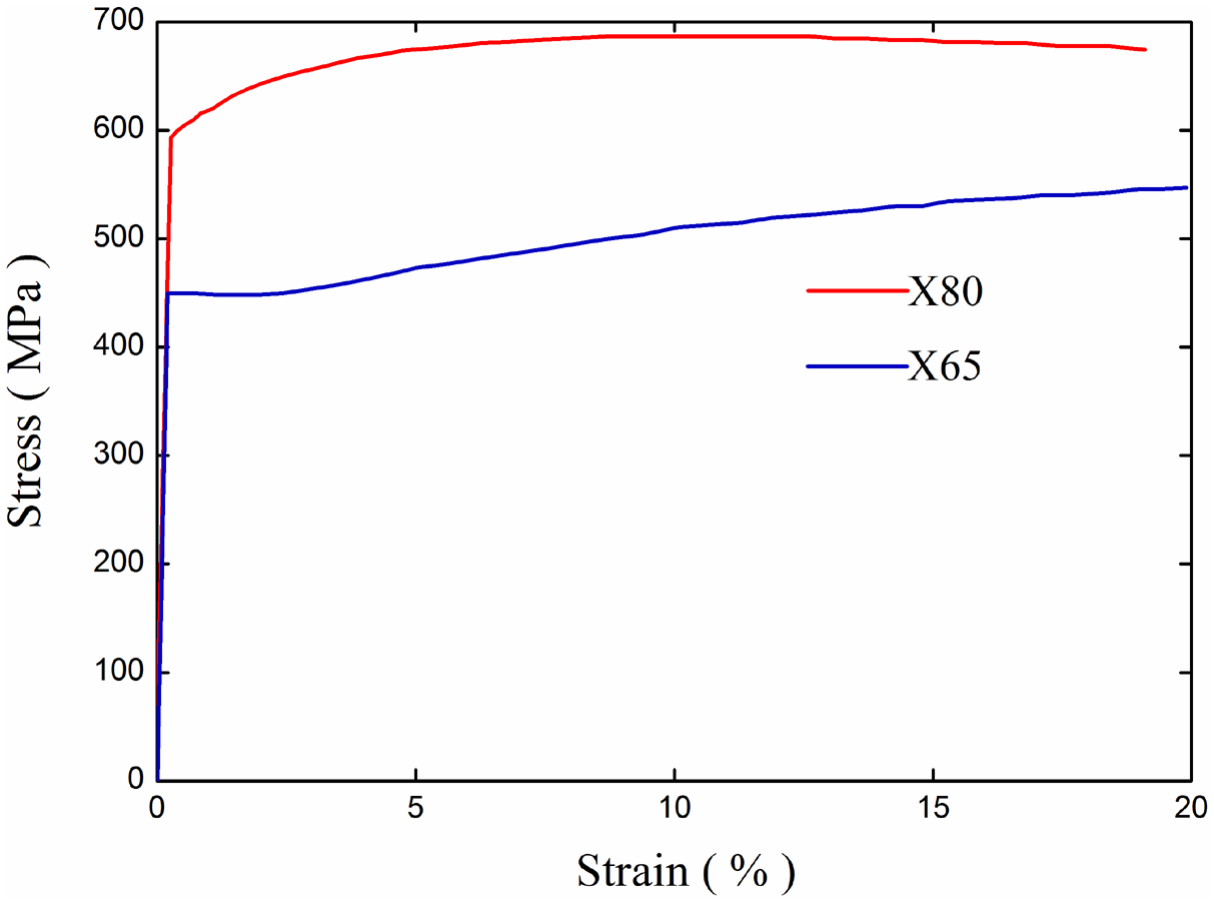
Stress-strain curves of X65 and X80. Relationship of stress and strain of the two buried pipeline materials.

Taking loess for example, it has a cohesion *c* = 24.6kPa, friction angle *ϕ* = 11.7° [17], Young's modulus *E* = 33MPa, Poisson's ratio *ν* = 0.44, density *ρ* = 1400 kg/m^3^. X65 and X80 are typical steel materials for oil and gas pipeline application, with a nominal stress-strain curve shown in Fig 3 [2]. The yield stress *σ*_y_ of X65 and X80 are 448.5MPa and 596MPa respectively. Young's modulus of the steel material equal to 210GPa, Poisson's ratio is 0.3, density is 7800kg/m^3^. Considering a safety factor equal to 0.72, as suggested in [18], and the maximum operating pressure *P*_max_ of this pipeline, given by the following expression *P*_max_ = 0.72×(2*σ*_*y*_ *t*/*D*).

**Fig 4:**
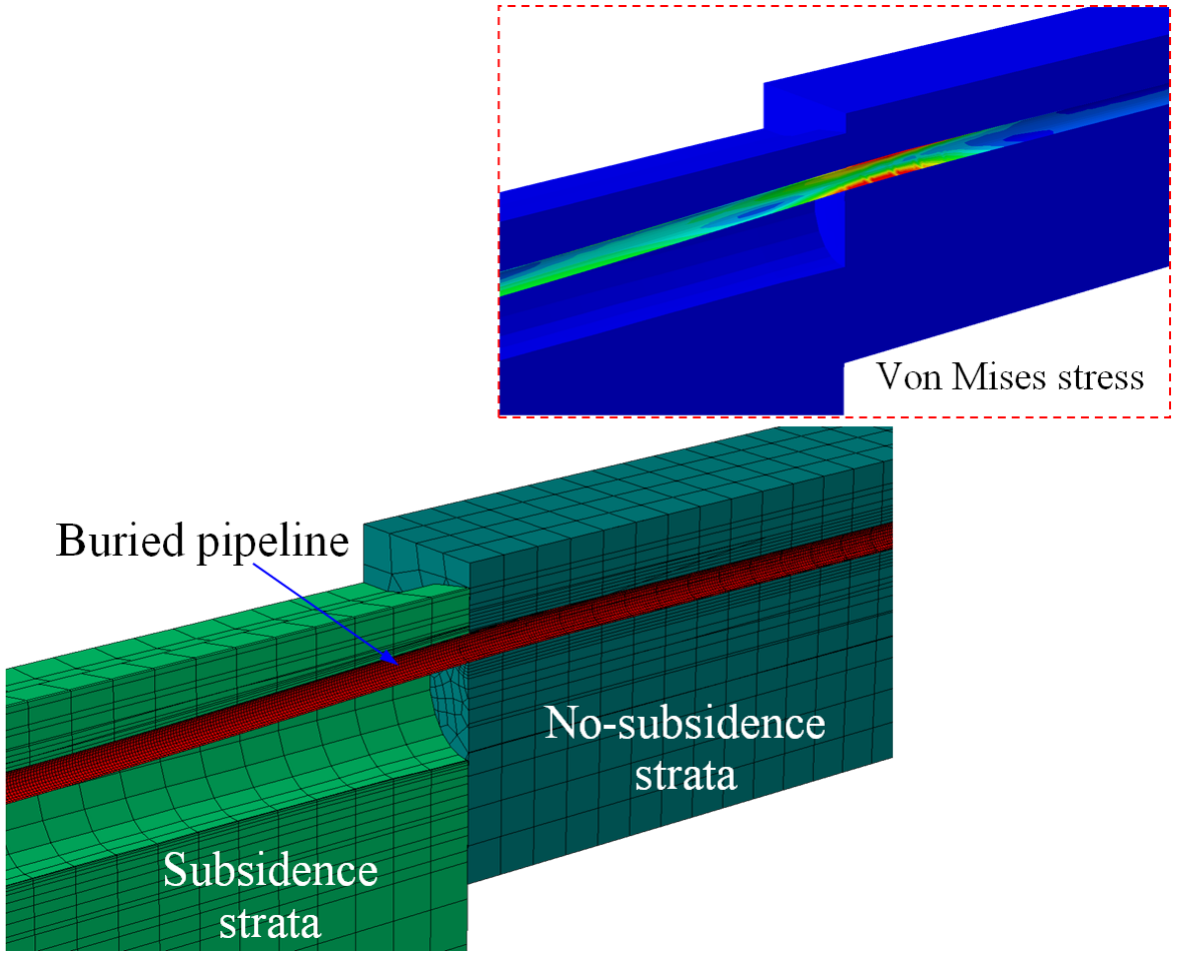
Deformations of the strata and buried pipeline. Deformations of the hole wall in the subsidence strata and no-subsidence strata are different.

Fig 4 shows the deformations of the strata and buried pipeline. Bending deformation of the pipeline occurs under the strata subsidence. Deformation of the no-subsidence strata is small. While the deformation of the subsidence strata is bigger. There is a bigger deformation for the hole wall, and swell appears on the surface of subsidence strata. In the subsidence strata, the buried pipeline only contact with the upper part of the hole wall. The maximum von Mises stress appears on the pipeline in outer transition subsidence area firstly.

**Fig 5:**
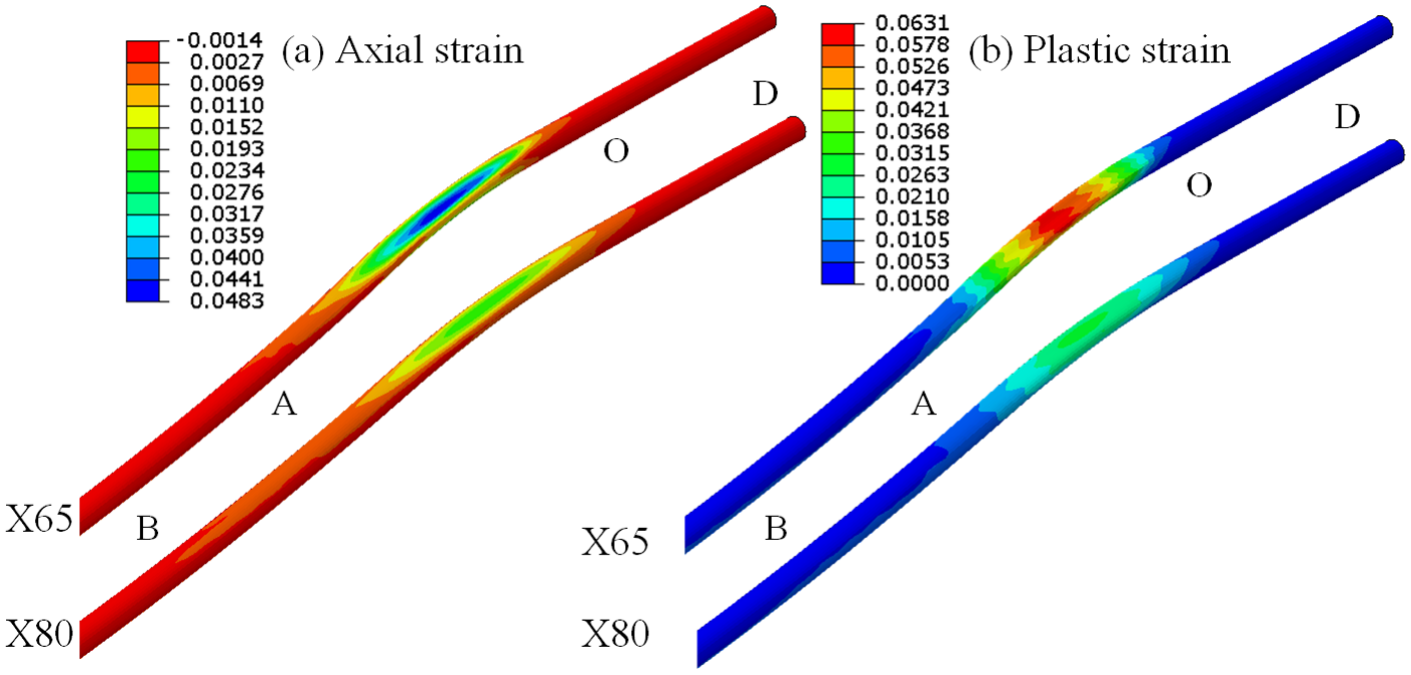
Longitudinal strain and equivalent plastic strain of DA pipeline. (a) Longitudinal strain of DA section pipeline. (b) Equivalent plastic strain of DA section pipeline.

At a value of the ratio between subsidence displacement and pipeline diameter equal to 8.75, longitudinal strain and equivalent plastic strain of DA pipeline are shown in Fig 5. Longitudinal strain describes the strain in *x* direction. Equivalent plastic strain describes the accumulation of plastic strain in the whole deformation process. The strain of OA pipeline is the biggest for the whole pipeline. The maximum longitudinal strain of X80 is 0.0288, while X65 is 0.0483. So, the ratio of them is 1:1.68. The maximum equivalent plastic strain of X80 is 0.028. It decreased 1.25 times than X65, whose maximum equivalent plastic strain is 0.063. From the simulation results, mechanical performance of X80 is preferable to X65. There is a little failure probability for X80 under the same conditions, and high quality steel pipeline material can better resist bucking and other failure caused by external load.

As shown in Fig 5a, longitudinal strains of pipeline present elliptic distribution, the maximum strain appears in the middle of the pipeline. Along radial direction of the elliptic distribution, the longitudinal strain reduces gradually. Larger cave appears on the bigger longitudinal strain. Because of the OA segment pipeline under outer subsidence area, the pipeline presents a convex shape. So, the upper part of the pipeline is in tension and the lower part in compression.

### Sensitivity parameters analysis

The mechanical properties of buried pipelines under subsidence strata are influenced by ground subsidence, diameter-thickness ratio, internal pressure, buried depth and pipeline-soil friction coefficient. Thus, research on the effects of sensitivity parameters on mechanical behavior of buried pipeline can provide a theoretical basis for the laying design, restoration and protection.

### Strata subsidence

**Fig 6:**
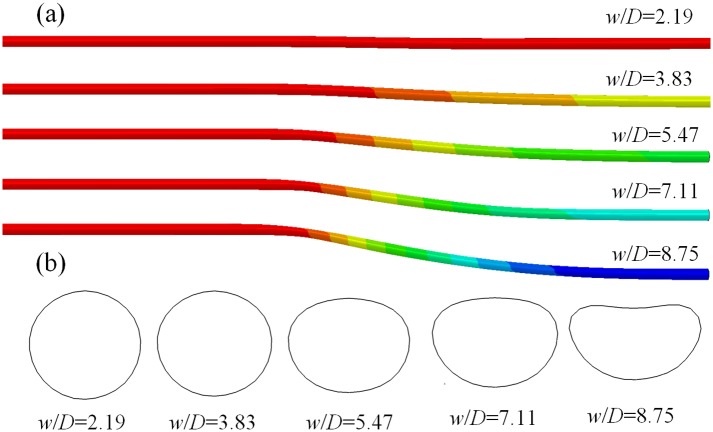
Bending deformations and cross section shapes under different subsidence. (a) Bending deformation of the buried pipeline. (b) Cross section shape of the most dangerous part of the buried pipeline.

When strata subsidence is small, buried pipeline will be subsidence with the strata at the same time, and bending deformation appears. When the strata subsidence is too large, pipeline may be pulled cut or formed a dangling. Fig 6 shows the deformation and the most dangerous cross section shapes of buried pipeline under different strata subsidence *w* for X65. As shown in Fig 6a, bending deformation of OA segment pipeline is the biggest, failure will be first occurred in this segment. Bending deformation of the buried pipeline increases with the increasing of strata subsidence *w*. As shown in Fig 6b, with the increasing of strata subsidence, the pipeline section shape of the most dangerous segment from a circular to an ellipse gradually, then to a crescent. For the cross section of pipeline, compression deformation of the lower part is small, while tensile deformation of the upper part is bigger, then the sunk is formed.

**Fig 7:**
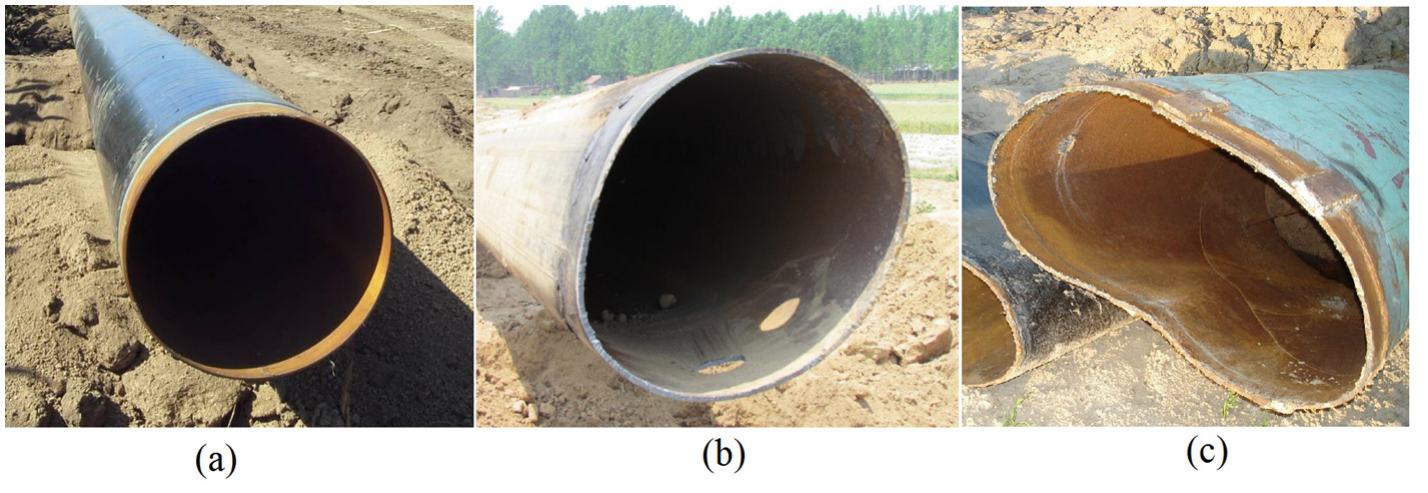
Cross section shapes of the pipeline before and after failure. (a) Circular cross section of the new pipeline. (b) Elliptical cross section. (c) Crescent cross section.

Cross section shapes of the pipeline before and after failure are shown in Fig 7. New pipeline with circular cross section (as shown in Fig 7a), cross section of pipeline becomes elliptical after small strata subsidence (as shown in Fig 7b), and crescent cross section would be appear if strata subsidence is bigger (as shown in Fig 7c). Irregular cross section of the pipeline is not conducive for the pigging job, and it is easy to cause leakage of oil and gas when pipeline ruptures.

**Fig 8:**
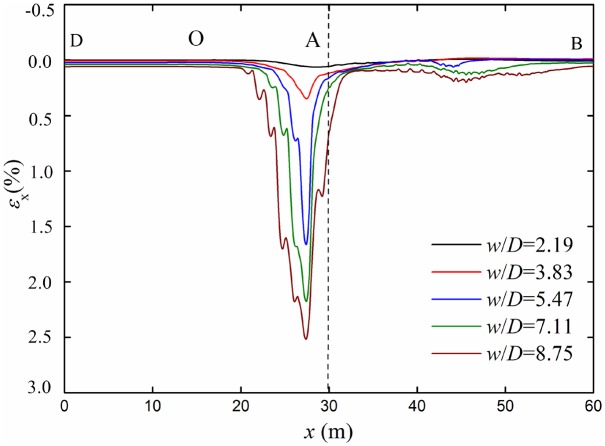
Longitudinal strain curves under different subsidence. Dotted line represents the boundary between the settlement and no-settlement strata.

Fig 8 shows the longitudinal strain curves of the top point in the pipeline cross section under different strata subsidences. Along the axial direction of the pipeline, a peak of longitudinal strain curve appears in the OA segment. The maximum strain is not on the dislocation point (point A) of the two blocks, but in the location about 2.5m from point A. Longitudinal strain of pipeline increases with the increasing of the strata subsidence. Longitudinal strain of OD segment pipeline is very small for no subsidence. Longitudinal strain of AB segment pipeline increases first and then decreases along AB direction.

**Fig 9:**
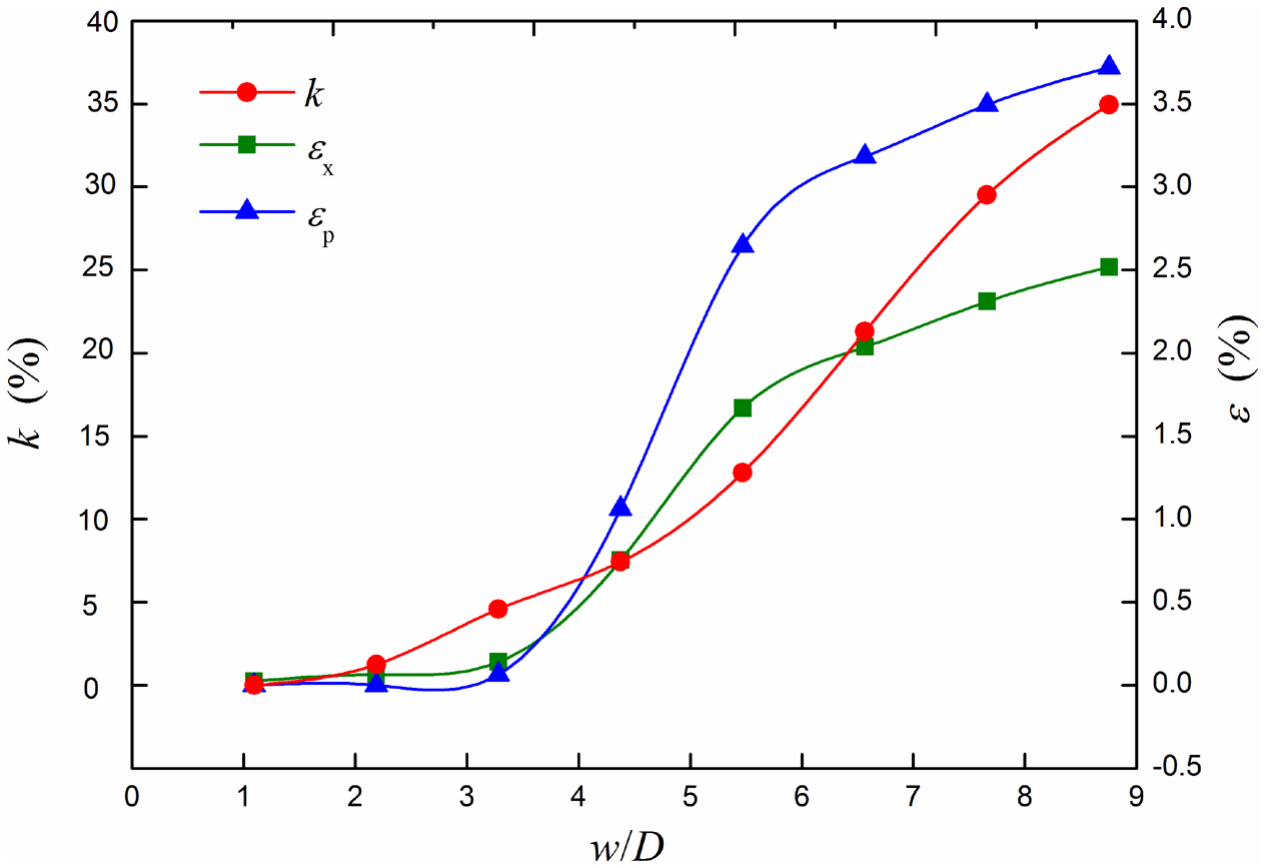
Out of roundness, longitudinal strain and equivalent plastic strain curves under different subsidence. Out of roundness *k*, longitudinal strain *ε*_x_ and equivalent plastic strain *ε*_p_ change with the increasing of strata subsidence.

In order to describe the cross section shape of deformed pipeline, out-of-roundness *k* is defined (*k* = (*D*_max_−*D*_min_)/*D*). The most dangerous section out-of-roundness, the maximum longitudinal strain and equivalent plastic strain curves under different strata subsidence are shown in Fig 9. The most dangerous section out-of-roundness *k*, the maximum longitudinal strain *ε*_x_ and equivalent plastic strain *ε*_p_ increase with the increasing of the subsidence with nonlinear rule. When the ratio of subsidence and diameter *w*/*D*<2, the equivalent plastic strain of the pipeline is zero. It means that the pipeline material is not yielding. In elastic state, the section out-of-roundness and elastic strain are smaller, but their growth rate gradually increases first and then decreases in the plastic state. It is dangerous for pits occurred in the longitudinal welding seam position in API579 standards, because it is very to generate crack [19]. Some think that pits on welding seam position will greatly reduce the rupture strength of the pipeline.

### Diameter-thickness ratio

Diameter-thickness ratio influences the load capacity and ability of resistance to deformation. The outer diameter *D* of the pipeline is equal to 914.4mm, five values for the pipeline wall thickness are considered, namely 6.5mm, 10mm, 13.5mm, 17mm and 20.5mm, corresponding to *D*/*t* values equal to 140.6, 91.4, 67.7, 53.8 and 44.6 respectively, which cover a wide range of oil and gas pipeline applications.

**Fig 10:**
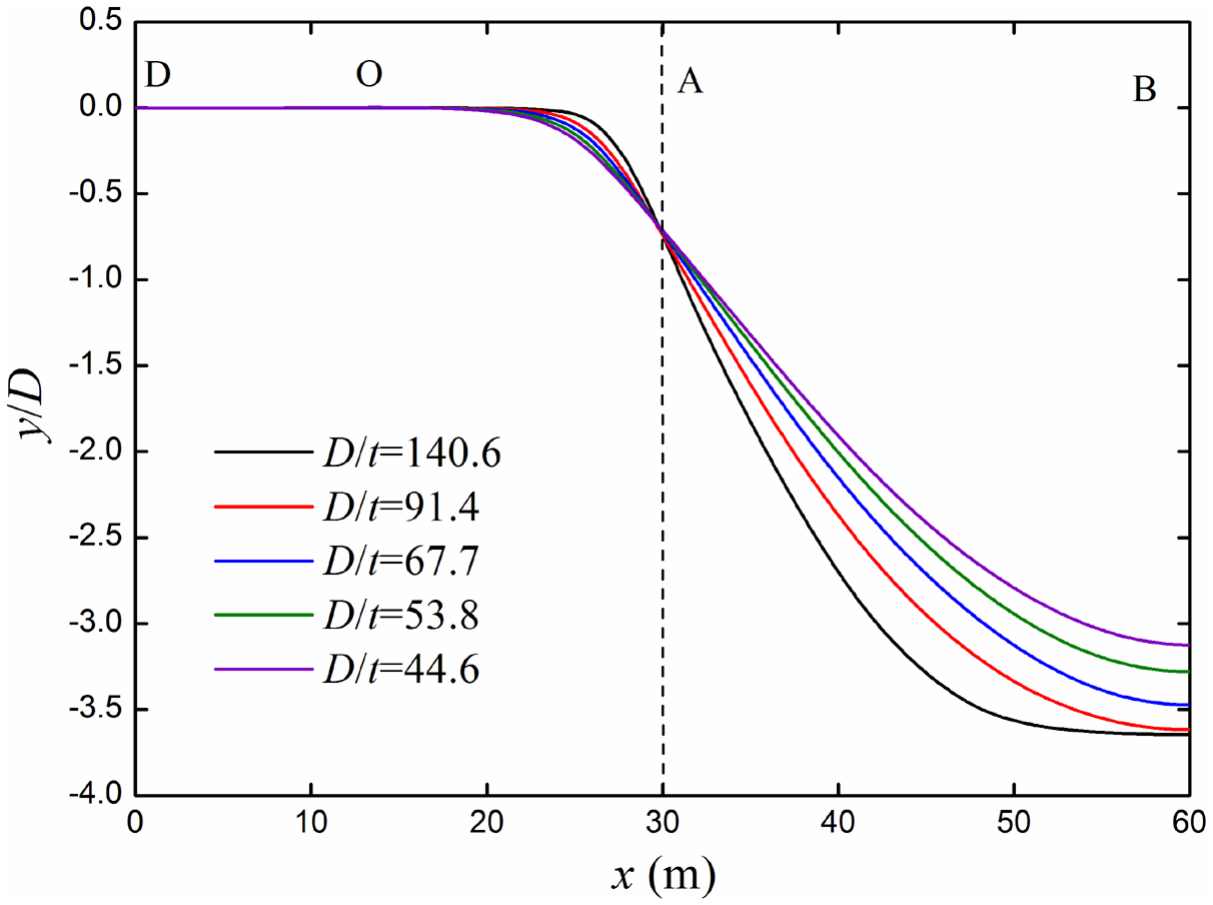
Deflection curves of buried pipelines under different diameter-thickness ratios. The deflection curve comes from the displacement of the nodes in the neutral surface for the buried pipeline.

Considering *w*/*D* is equal to 8.75, the deflection curves of buried pipeline under different diameter-thickness ratios are shown in Fig 10. No matter any value of diameter-thickness ratio, bending deformation of point A remains the same, which illustrates that the subsidence of dislocation point of the two blocks has nothing to do with the diameter-thickness ratio. Subsidence of AB segment pipeline increases gradually with the increasing of diameter-thickness ratio. While the radius of curvature of OA segment pipeline decreases with the increasing of diameter-thickness ratio, which means the bending deformation of OA segment pipeline is bigger, it is easy to failure. Therefore, the resistance ability to deformation of buried pipeline decreases with the increasing of diameter-thickness ratio.

**Fig 11:**
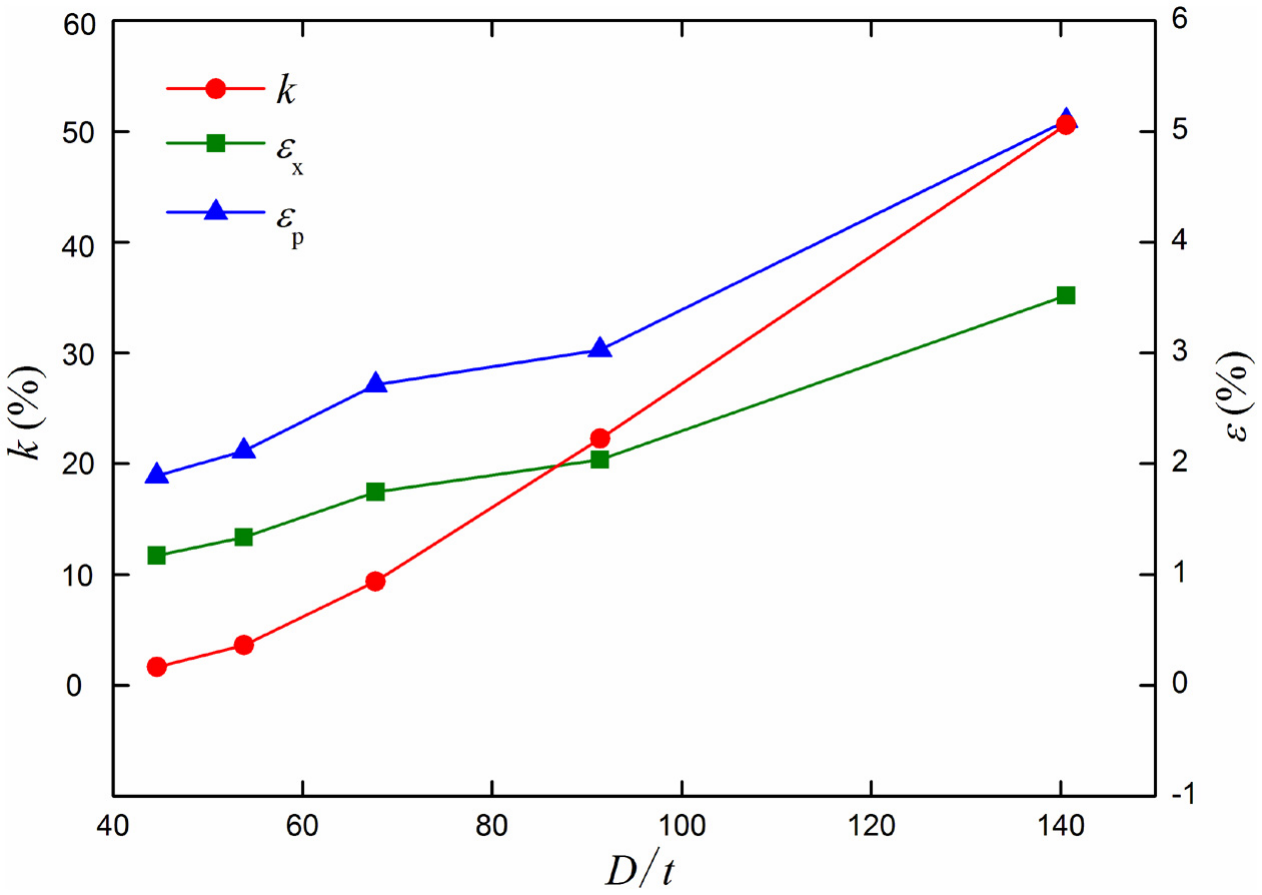
Out of roundness, longitudinal strain and equivalent plastic strain curves under different diameter-thickness ratios. Diameter-thickness ratio *D*/*t* affects the bearing capacity and mechanical strength of the pipeline.

The most dangerous section out-of-roundness *k*, the maximum longitudinal strain *ε*_x_ and equivalent plastic strain *ε*_p_ curves under different diameter-thickness ratios are shown in Fig 11. With the increasing of diameter-thickness ratio *D*/*t*, section out-of-roundness *k*, the maximum longitudinal strain *ε*_x_ and equivalent plastic strain *ε*_p_ increase with nonlinear rule. The change of section out-of-roundness *k* increases also. The stain curve rules of the maximum longitudinal strain *ε*_x_ and equivalent plastic strain *ε*_p_ are more similar.

### Buried depth

**Fig 12:**
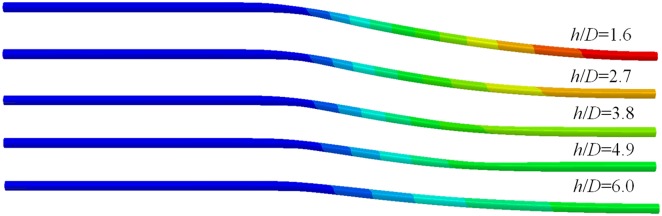
Bending deformation of buried pipelines under different buried depths. Deformation of buried pipeline decreases with the increasing of buried depths.

**Fig 13:**
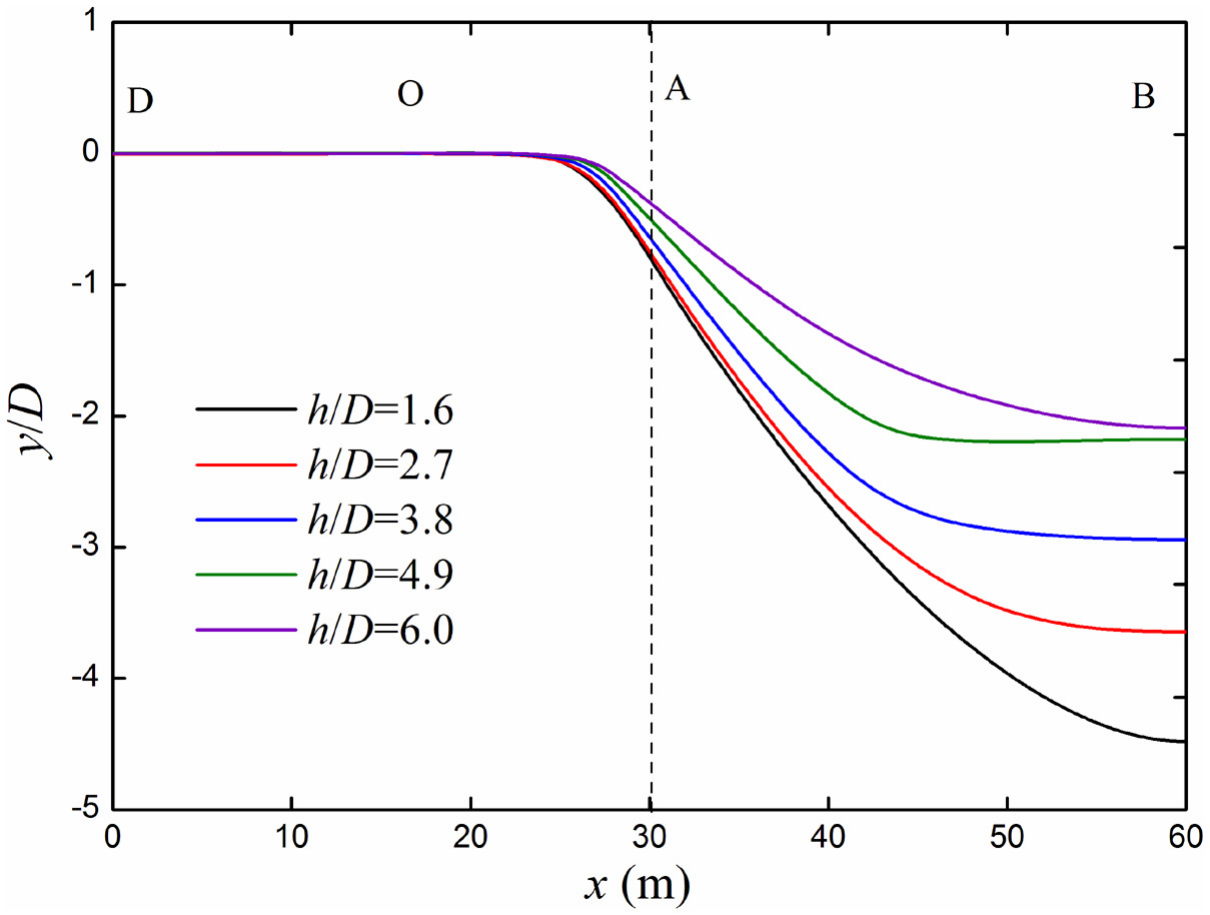
Deflection curves of buried pipelines under different buried depths. Buried depth has a great effect on the buried pipeline in subsidence strata, but has a small effect on the other section.

The bending deformation and deflection curves of different ratios of buried depth to diameter *h*/*D* are shown in Figs 12 and 13. Bending deformation decreases with the increasing of the buried depth. When strata subsidence occurs, all parts of the pipeline not only overcome the compressive or tensile stress from adjacent parts and friction force with the surrounding soil, but also need to resistance the subsidence of overlying soil. When the buried depth of pipeline is shallow, the thinner the thickness of overlying soil, the smaller the force of its on the pipeline is. If the gravity and cohesive force of overlying soil are not enough to overcome the resistance of pipeline, the deformation of formation pore wall will pass to strata surface. Then the strata's surface uplift appears. When the thickness of the overlying soil is thicker, deformation of formation pore wall will not pass to strata surface. According to Hooker theorem, the hole wall deformation caused from pipeline increase with the increasing of the overlying soil. The upper part of the hole wall contact with the pipeline in the strata subsidence, therefore, the bending formation of pipeline decreases with the increasing of the buried depth.

**Fig 14:**
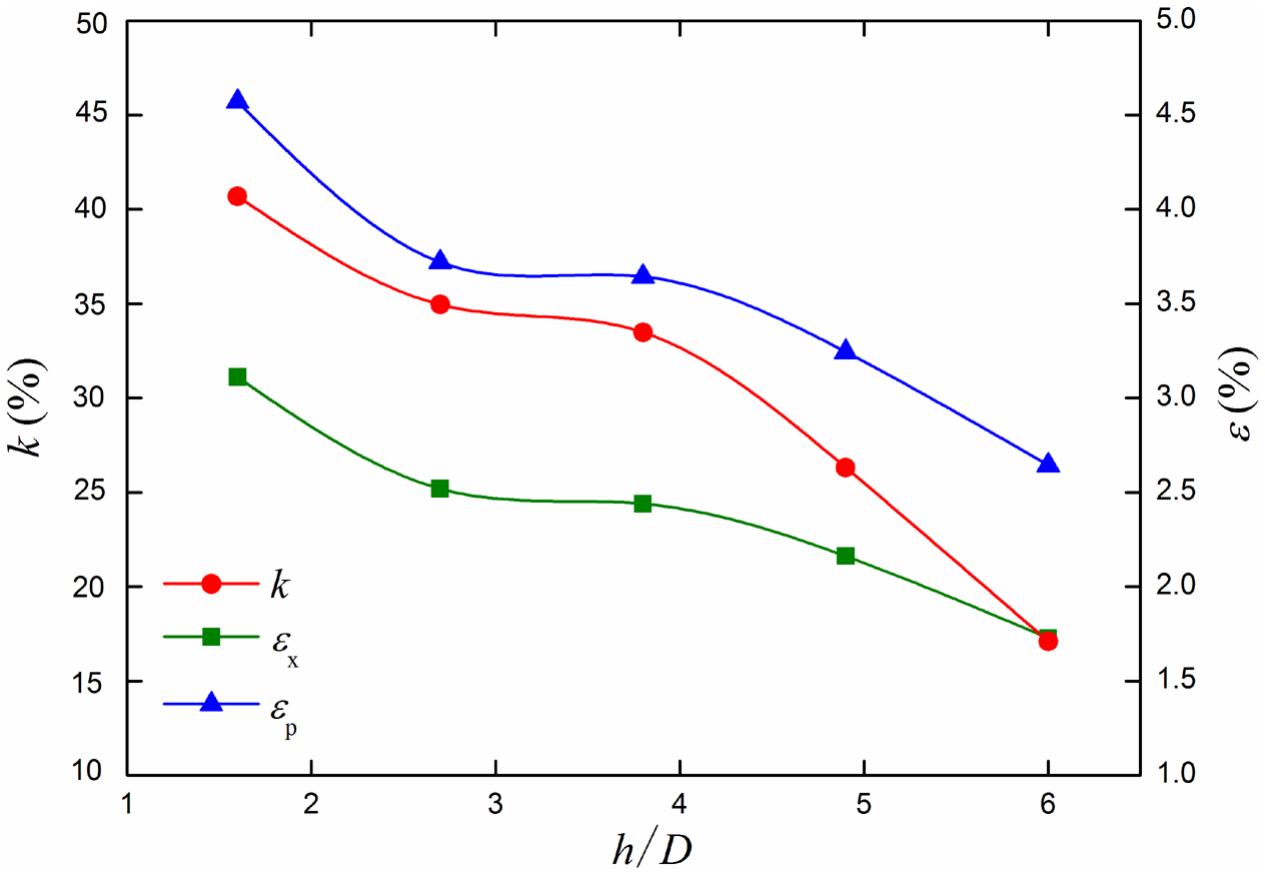
Out of roundness, longitudinal strain and equivalent plastic strain curves under different buried depths. Out of roundness *k*, longitudinal strain *ε*_x_ and equivalent plastic strain *ε*_p_ change with the increasing of the buried depth.

The most dangerous section out-of-roundness *k*, the maximum longitudinal strain *ε*_x_ and equivalent plastic strain *ε*_p_ curves under different ratio of buried depth to diameter are shown in Fig 14. With the increasing of the ratio of buried depth to diameter *h*/*D*, the curves of section out-of-roundness *k*, the maximum longitudinal strain *ε*_x_ and equivalent plastic strain *ε*_p_ decrease, and change rules of all values are similar.

### Internal pressure of pipeline

**Fig 15:**
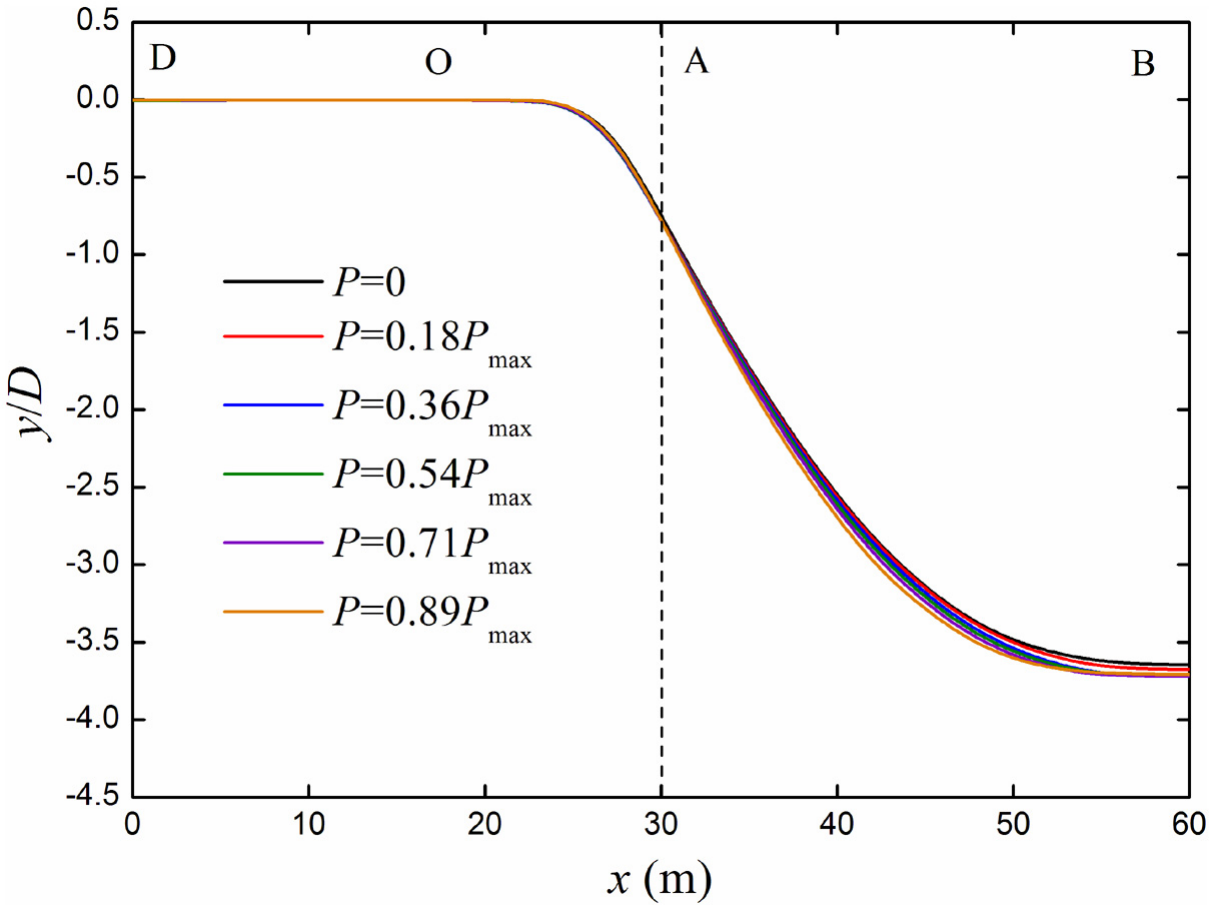
Deflection curves of buried pipelines under different internal pressures. Buried pipeline with different pressures (0~ *P*_max_) was investigated.

The deflection curves of pipeline under different internal pressures are shown in Fig 15. With the scope of the allowable maximum internal pressure *P*_max_, no matter the internal pressure is, bending deformation of the buried pipeline is the same as the no internal pressure condition. It illustrates that the internal pressure does not influence the bending deformation of the pipeline.

**Fig 16:**
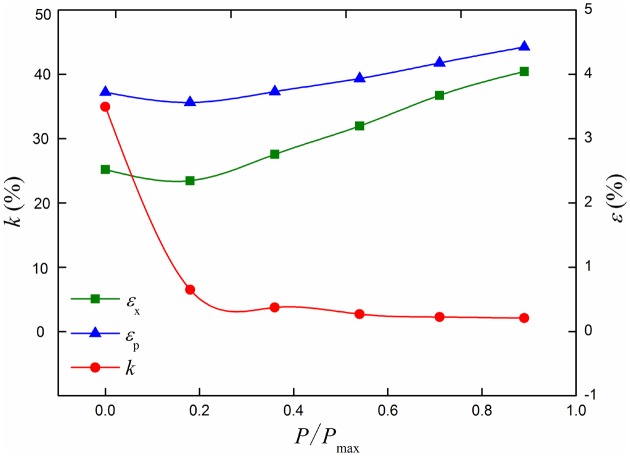
Out of roundness, longitudinal strain and equivalent plastic strain curves under different internal pressures. Internal pressure can enhance the ability of resistance to deformation for the buried pipeline, out of roundness *k*, longitudinal strain *ε*_x_ and equivalent plastic strain *ε*_p_ can be affected by the internal pressure.

The most dangerous section out-of-roundness *k*, the maximum longitudinal strain *ε*_x_ and equivalent plastic strain *ε*_p_ curves under different internal pressures are shown in Fig 16. With the increasing of internal pressure, the maximum longitudinal strain *ε*_x_ and equivalent plastic strain *ε*_p_ of buried pressure pipeline increases gradually. Section out-of-roundness *k* of no-pressure pipeline is bigger than pressure pipeline. And it decreases with the increasing of the internal pressure for pressure pipeline. When *P*/*P*_max_<0.36, the change rate of *k* is bigger than the condition of *P*/*P*_max_>0.36.

### Friction coefficient

In the process of strata subsidence, the friction force between surrounding soil and pipeline can be divided into two parts. One is static friction force before the soil yield, and the other is the sliding friction force after the soil yield [15]. When the axial deformation of the pipeline appears, the surrounding soil will be resistance to the relative movement. When the resistance reaches limit value, the surrounding soil will yield. Then relative sliding appears between pipeline and soil.

Considering friction coefficient *f* equal to 0.2~0.6, the most dangerous section out-of-roundness *k*, the maximum longitudinal strain *ε*_x_ and equivalent plastic strain *ε*_p_ are shown in Table 1. Pipeline settlement changes are small under different friction coefficients, so effect of friction coefficient *f* on bending deformation of pipeline is small. Section out-of-roundness *k* increases with the increasing of friction coefficient *f*, but when *f* >0.4, the change rate is very small. With the increasing of friction coefficient *f*, the maximum longitudinal strain *ε*_x_ and equivalent plastic strain *ε*_p_ increase with a small growth rate.

**Table 1:** Results under different friction coefficients.

*f*	*y*/*D*	*k*(%)	*ε*_p_(%)	*ε*_x_(%)
0.2	3.990	33.65	3.642	2.480
0.3	3.988	34.96	3.720	2.519
0.4	4.006	35.55	3.818	2.574
0.5	3.994	35.80	3.875	2.605
0.6	3.992	35.68	3.879	2.609

The maximum displacement *y*, out of roundness *k*, longitudinal strain *ε*_x_ and equivalent plastic strain *ε*_p_ of the buried pipeline were calculated under different friction coefficients.

### Different soils

Deformations of buried pipeline are different under different soils. Considering loess, sand and clay, the bending deformation and strain were analyzed, the soil physical parameters of sand and clay are shown in Table 2.

**Table 2:** Physical parameters of soils.

Strata	*c* (kPa)	*ϕ* (°)	*E*(MPa)	*ν*	*ρ*(kg/m^3^)
Sand [20]	5.66	35.19	20.35	0.35	2013
Clay [21]	47.0	34.0	18	0.35	1960

Sand and clay are also described through an elastic-perfectly plastic Mohr-Coulomb constitutive model, characterized by cohesion *c*, friction angle *ϕ*, elastic modulus *E*, and Poisson's ratio *ν*.

**Fig 17:**
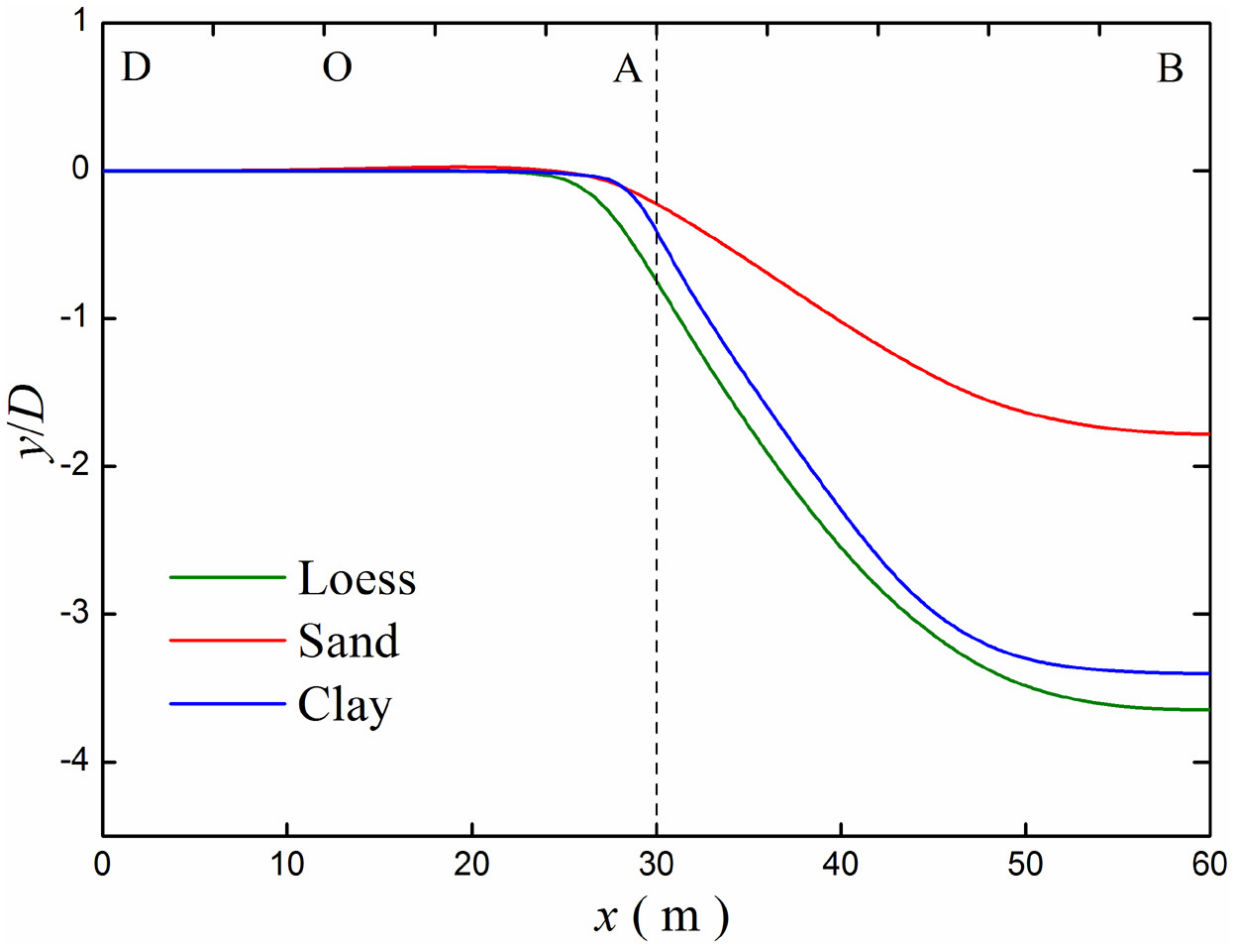
Deflection curves of buried pipelines under different soils. Buried pipelines under loess strata, sand strata and clay strata were investigated.

The deflection curves of pipeline under different soils are shown in Fig 17. Bending deformation of the pipeline in loess strata is the biggest, while it is the smallest in sand strata for the deformation of sand strata is bigger under the load of the pipeline.

Considering different soils, the most dangerous section out-of-roundness *k*, the maximum longitudinal strain *ε*_x_ and equivalent plastic strain *ε*_p_ are shown in Table 3. Section out-of-roundness *k*, the maximum longitudinal strain *ε*_x_ and equivalent plastic strain *ε*_p_ are the smallest in sand strata. Although the bending deformation of pipeline in loess strata is bigger than in clay strata (as shown in Fig 17), the section out-of-roundness *k*, the maximum longitudinal strain *ε*_x_ and equivalent plastic strain *ε*_p_ are smaller. Therefore, deformation and strain of pipeline have a great relationship with the physical properties of surrounding soil.

**Table 3:** Pipeline strains under different soils.

Strata	*k*(%)	*ε*_p_(%)	*ε*_x_(%)
Loess	34.96	3.720	2.519
Sand	10.79	0.944	0.700
Clay	37.17	5.555	3.799

Out of roundness *k*, longitudinal strain *ε*_x_ and equivalent plastic strain *ε*_p_ of the buried pipeline under loess, sand and clay stratums are shown in the table.

## Conclusions

1. Using advanced finite element simulation tools, the mechanical behavior of buried steel pipeline crossing subsidence strata was investigated. The mechanical model of buried pipeline was established. The longitudinal strain of buried pipeline presents elliptic distribution, pipeline sections shape of the most dangerous segment from circular to ellipse gradually, then to crescent with the increasing of strata subsidence.
2. The section out-of-roundness, the maximum longitudinal strain and equivalent plastic strain increase with the increasing of strata subsidence and diameter-thickness ratio of pipeline. With the increasing of buried depth, bending deformation, out-of-roundness, longitudinal strain and equivalent plastic strain decrease. Internal pressure has a little effect on the bending deformation of pipeline, but out-of-roundness decreases while the strain increases with the increasing of internal pressure. The bending deformation and strain of pipeline under different soils are very different.
